# Mechanistic Insights into Regulation of the Aryl Hydrocarbon Receptor Expression in Mouse and Human Hepatoma Cells

**DOI:** 10.3390/ijms27188369

**Published:** 2026-09-20

**Authors:** Yiyuan Wang, William K. Chan

**Affiliations:** Department of Pharmaceutical Sciences, Thomas J. Long School of Pharmacy, University of the Pacific, Stockton, CA 95211, USA; y_wang48@u.pacific.edu

**Keywords:** aryl hydrocarbon receptor, AHR, proteasomal degradation, regulation of AHR expression, (S)-MG132, Hepa1c1c7, HepG2, Hep3B

## Abstract

The aryl hydrocarbon receptor (AHR) is a ligand-activated transcription factor which involves a myriad of cellular functions ranging from xenobiotic sensing in the liver to immune response modulation. Discovery of endogenous tryptophan metabolites as ligands of this receptor allows us to understand the roles of AHR in cell growth and immune responses, making this receptor an attractive target for treatment of cancer and autoimmune diseases. Much emphasis has been focused on the involvement of AHR in cellular functions; however, mechanisms that affect the cellular expression of this receptor are poorly understood. Historically, our knowledge of AHR has been primarily acquired from the mouse Hepa1c1c7 hepatoma cells because this receptor is robustly expressed and functional in these cells. Since AHR is also active in human liver, human hepatoma cell lines such as Hep3B and HepG2 have been widely used to study AHR function. In this paper, we investigate how the AHR protein levels are maintained in these hepatoma cells. We provide mechanistic insights into the regulation of AHR expression using the HaloTag fusion of mouse AHR, together with two-dimensional (2D) monolayer and three-dimensional (3D) spheroid culture models. In summary, we prove that AHR is continuously degraded by proteasomal degradation without exogenous ligands in hepatoma monolayer and spheroids. AHR in mouse Hepa1c1c7, but not human Hep3B and HepG2, cells also undergo lysosomal degradation to a lesser extent. Inhibition of proteasomal degradation blocks the degradation and synthesis of the AHR protein.

## 1. Introduction

The aryl hydrocarbon receptor (AHR) has diverse functions in humans. When AHR was discovered in the 1970s, its function was limited to our response to xenobiotics, which was primarily related to its action in the liver [1]. Nowadays, AHR has been implicated in seemingly unrelated physiological functions and diseases, namely liver homeostasis such as hepatic fibrosis [2] and alcohol-related liver disease [3]; skin homeostasis such as skin barrier dysfunction [4]; allergic rhinitis [5]; autoimmune diseases such as ulcerative colitis [6] and multiple sclerosis [7,8]; bacterial and viral infection [9,10,11]; uremic cardiomyopathy [12]; cancers of breast [13,14,15], lung [16], pancreas [17], brain [18], and gallbladder [19]; CNS axon regeneration [20]; cortical bone and skeletal muscle function [21,22]; eye diseases such as glaucoma [23] and macular degeneration [24]; diabetes [25]; and stem cell differentiation [26]. Researchers have unveiled many mechanisms via gene regulation and protein–protein interactions to explain various functions of AHR. However, fundamentally how cells regulate the AHR expression by controlling its synthesis and degradation is poorly understood.

Cellularly, AHR is a classical transcription factor that regulates gene transcription. Without activation, AHR resides in the cytoplasm as a complex containing HSP90, p23, XAP2 and c-Src [27,28]. The canonical signaling pathway is briefly summarized as follows: Binding of a ligand to AHR unveils the nuclear localization domain of AHR, allowing the whole complex to translocate into the nucleus. While in the nucleus, binding of ARNT with AHR dissociates the complex, allowing the formation of the AHR/ARNT heterodimer. This heterodimer acts as a transcription factor which recruits coactivators responsible for remodeling the local chromatin structure of the target genes, leading to upregulation of gene transcription. Some common AHR target genes include members of cytochrome P450 1A and 1B families (CYP1A1, CYP1A2, and CYP1B1), AHRR, and TiPARP [29]. Other than acting directly as a transcription factor, AHR is also a substrate protein that escorts client proteins—such as estrogen receptor, androgen receptor, and PPARγ—to Cullin4B E3 ligase for ubiquitination, followed by proteasomal degradation [30,31].

It is widely accepted that AHR undergoes proteasomal degradation after ligand activation. This degradation can be blocked by proteasome inhibitors such as MG132 [32]. Without ligand, however, it is unclear how cells regulate the AHR protein levels through protein synthesis and degradation. It appears that some human cell lines, such as cervical HeLa [33], lung A549 [34], and breast MDA-MB-468 [34] cells utilize various autophagic mechanisms (such as selective macroautophagy and chaperone-mediated autophagy) to degrade AHR in the absence of exogenous ligands, suggesting that AHR is continually degraded even without ligand activation.

In this study, we used mouse hepatoma (Hepa1c1c7) and human hepatoma (HepG2 and Hep3B) cells to investigate how hepatoma cells regulate AHR expression. Since there are variations of AHR protein degradation mechanisms which seem to be cell line-specific, we investigated how AHR protein levels are maintained in hepatoma cells to better understand how we can modulate AHR expression as a means of targeted therapy in the liver. Here, we provide evidence supporting that AHR undergoes proteasomal degradation in hepatoma cells without exogenous ligand treatment. Blockade of proteasomal degradation not only suppresses AHR from degradation but also affects AHR protein synthesis.

## 2. Results

### 2.1. Degradation of AHR Without Ligand Treatment in Mouse and Human Hepatoma Cells

**Figure 1:**
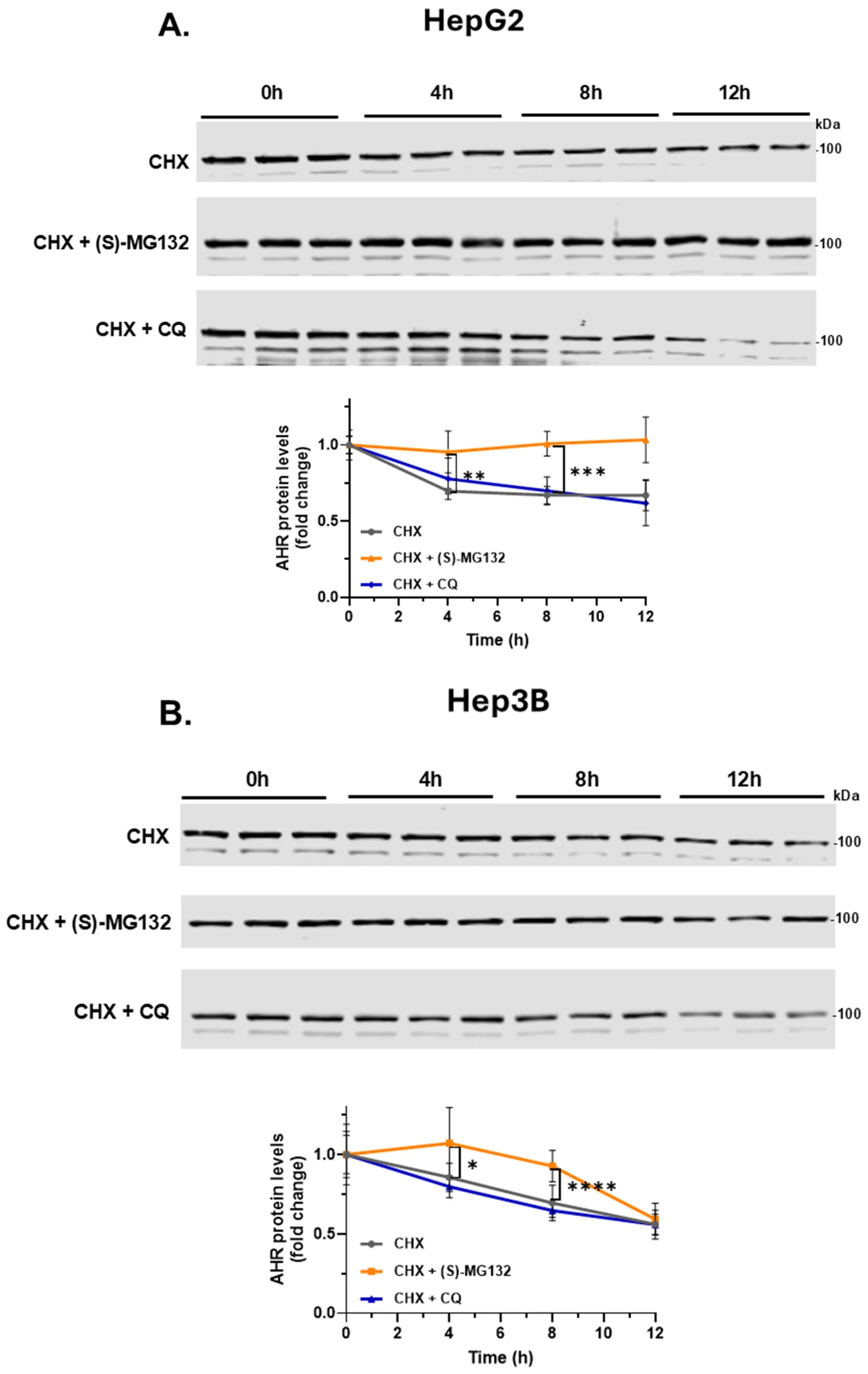

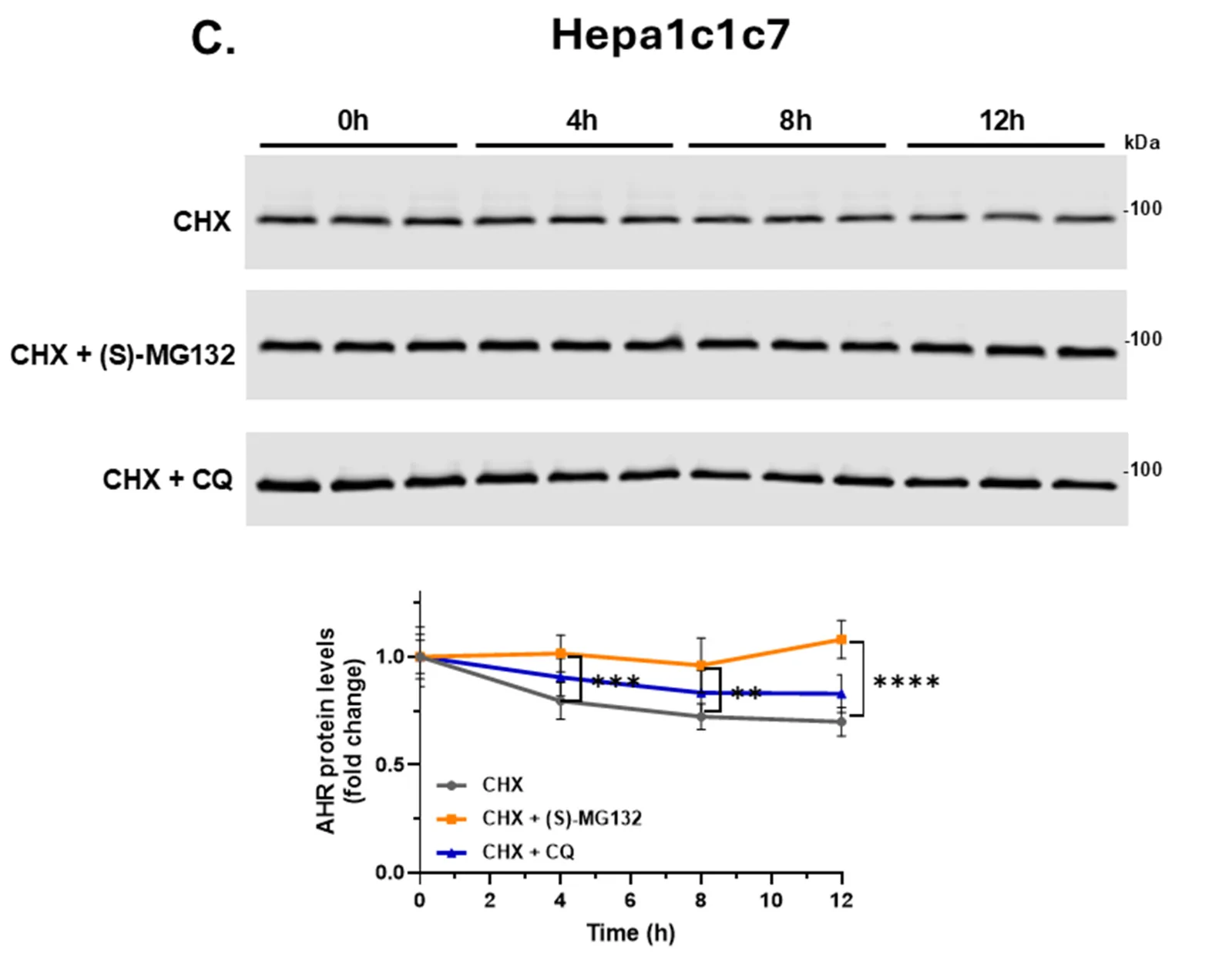
Basal AHR degradation in human and mouse hepatoma cells. AHR protein degradation was examined by CHX chase assay in HepG2 (**A**), Hep3B (**B**), and Hepa1c1c7 (**C**) cells. Cells were treated with CHX alone or in combination with the proteasome inhibitor (S)-MG132 or the lysosomal inhibitor CQ for 0, 4, 8, and 12 h at 37 °C. The treatment medium was exchanged with fresh medium containing the corresponding compounds at 4 and 8 h, and cells were harvested at each time point. HepG2 cells were treated with CHX (100 μg/mL), (S)-MG132 (10 μM), and CQ (100 μM). Hep3B cells were treated with CHX (50 μg/mL), (S)-MG132 (10 μM), and CQ (60 μM). Hepa1c1c7 cells were treated with CHX (50 μg/mL), (S)-MG132 (10 μM), and CQ (100 μM). AHR protein levels were determined by Western blot analysis and normalized to the corresponding 0 h time point. Representative Western blots and densitometric analyses are shown. Data are presented as means ± SD from at least six biological replicates (*n* ≥ 6) obtained from two independent experiments. Statistical significance was determined using one-way ANOVA followed by a multiple-comparisons test. Significance levels are indicated as *p* < 0.05 (*), *p* < 0.01 (**), *p* < 0.001 (***) and *p* < 0.0001 (****).

To assess whether AHR is continuously degraded via proteasomal or lysosomal degradation, or not, CHX, a protein synthesis inhibitor, was used to block global protein synthesis in the presence or absence of the proteasome inhibitor (S)-MG132 or the lysosomal inhibitor CQ. Results from the CHX chase assays revealed a modest decrease in AHR protein level over a 12 h period (t_1/2_ > 12 h) in all three cell lines (Figure 1A–C), suggesting that AHR is modestly degraded under basal conditions in hepatoma cells. In HepG2 cells, co-treatment with 10 µM (S)-MG132 clearly rescued AHR from degradation, whereas CQ did not alter AHR degradation (Figure 1A). In Hep3B cells, 10 µM (S)-MG132 stabilized AHR protein initially (up to 8 h), but this effect was not sustainable after 8 h. CQ did not affect the AHR degradation in Hep3B cells, which was similar to what we observed in HepG2 cells (Figure 1B). In Hepa1c1c7 cells, degradation of the AHR protein was significantly rescued, like HepG2, by 10 µM (S)-MG132. In addition, CQ showed a statistically significant rescue of AHR from degradation at 4 and 8 h after CHX treatment but not at 12 h (Figure 1C). Collectively, these findings supported that AHR is continually degraded via proteasomal degradation in human and mouse hepatoma cells without exogenous ligand treatment. Autophagy is not used to degrade AHR in human hepatoma cells but plays a lesser role in degrading AHR of mouse hepatoma cells.

### 2.2. Effect of (S)-MG132 on AHR mRNA and Protein Levels in Mouse and Human Hepatoma Cells

**Figure 2:**
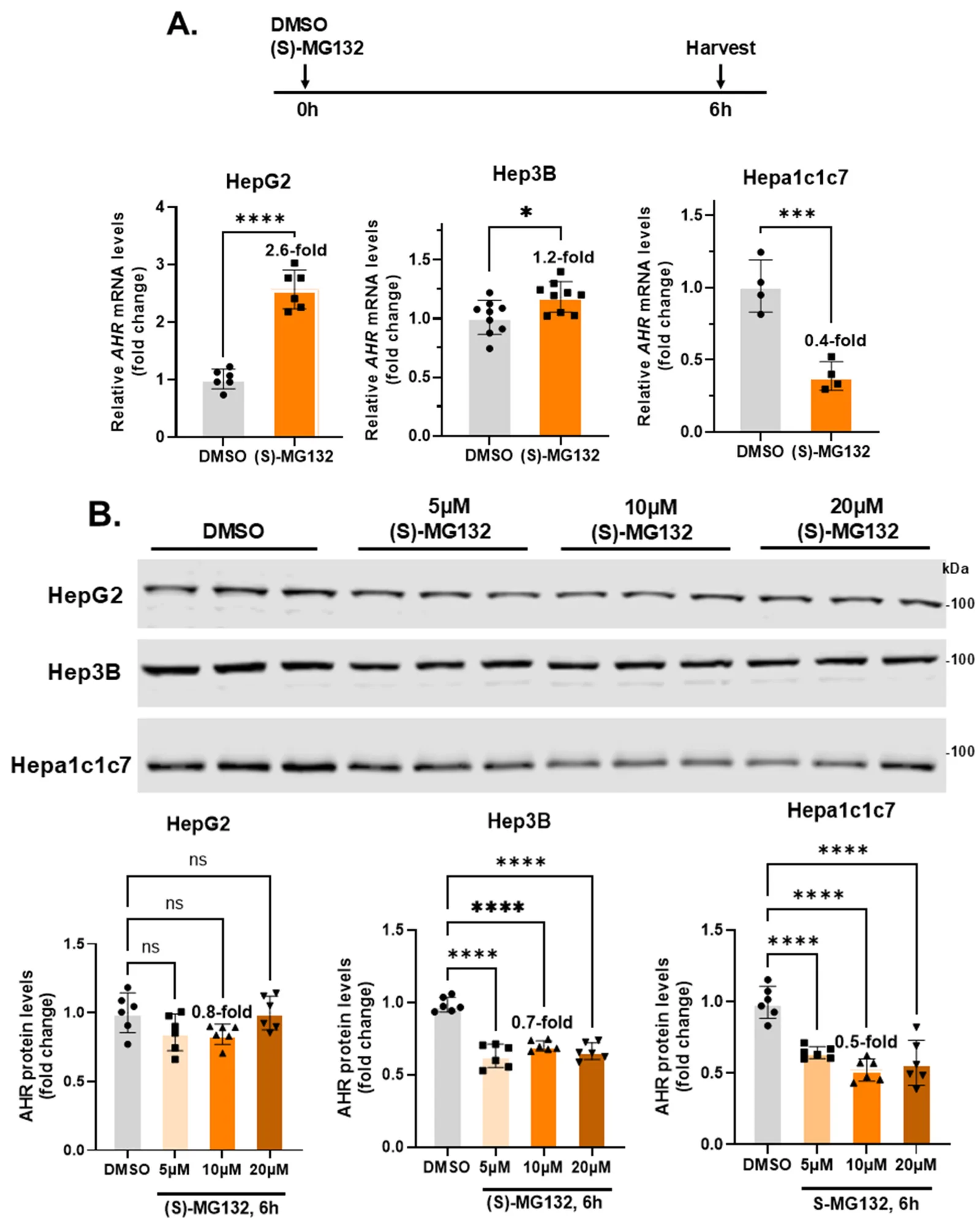

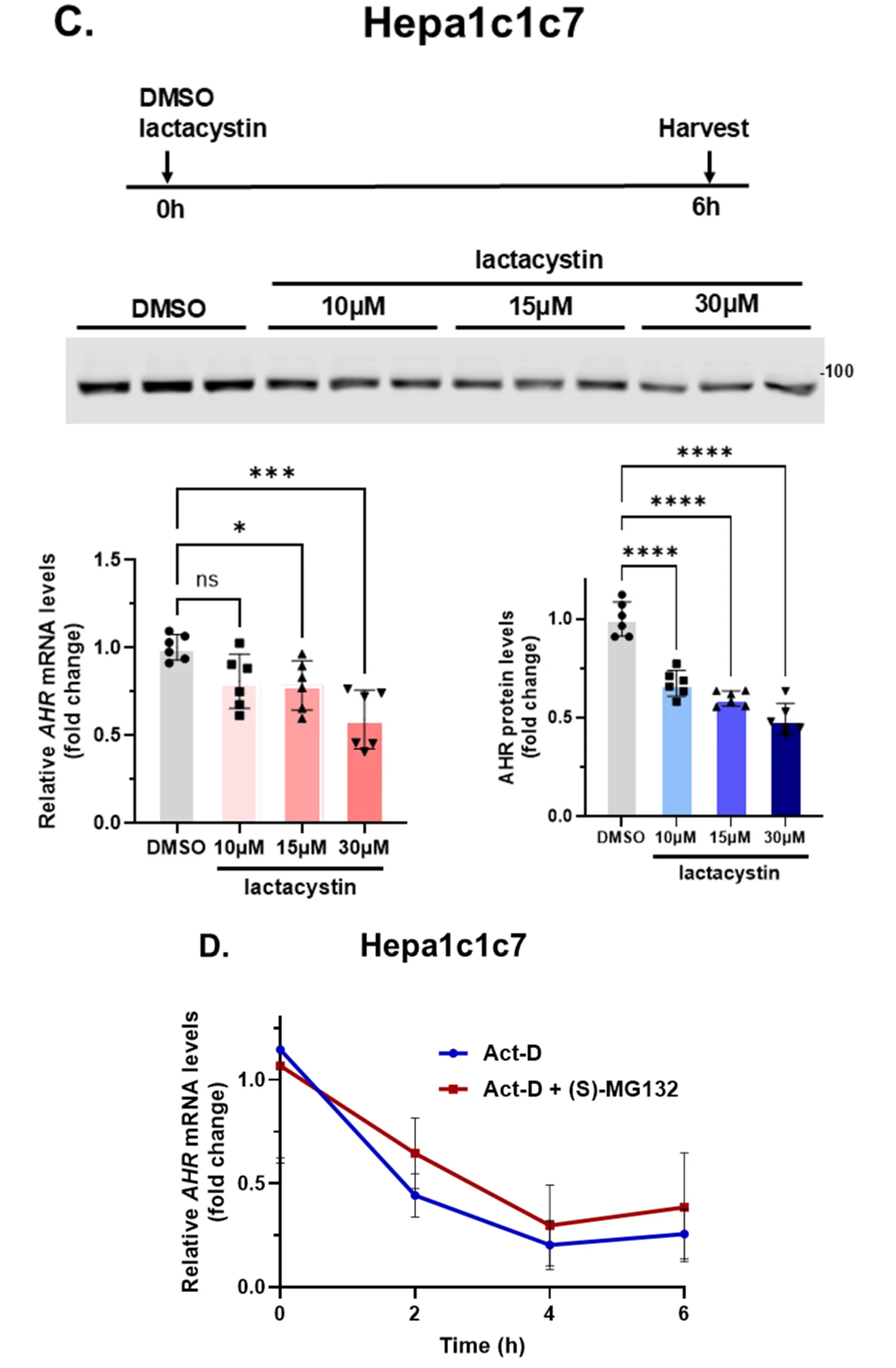
Effects of proteasomal inhibition on *AHR* mRNA and protein levels in human and mouse hepatoma cells. (**A**) Cells were treated with DMSO or 10 μM (S)-MG132 for 6 h at 37 °C. RT-qPCR results of *AHR* mRNA levels normalized to 18S rRNA (HepG2 and Hep3B) or Rps18 (Hepa1c1c7). Data are presented as fold change relative to DMSO-treated controls. (**B**) Cells were treated with increasing concentrations of (S)-MG132 (5, 10, and 20 μM) for 6 h at 37 °C. Western results of AHR protein levels normalized to DMSO-treated controls. (**C**) Hepa1c1c7 cells were treated with DMSO or increasing concentrations of lactacystin (10, 15, and 30 μM) for 6 h at 37 °C. RT-qPCR results of *AHR* mRNA levels first normalized to Rps18 and then normalized to DMSO group. Western results of AHR protein levels normalized to DMSO group. The DMSO group was arbitrarily set to 1. (**D**) Hepa1c1c7 cells were treated with actinomycin D (ActD, 5 µg/mL) in the presence or absence of 10 μM (S)-MG132 for the indicated times (0, 2, 4, and 6 h). Relative *AHR* mRNA levels were measured by RT-qPCR and normalized to Rps18. Data are presented as fold change relative to the zero time point. Data are presented as means ± SD. Statistical significance was determined using an unpaired two-tailed Student’s *t*-test (**A**), one-way ANOVA followed by a multiple-comparisons test (**B**,**C**), and two-way ANOVA (**D**). Each data point represents an independent biological replicate (*n* ≥ 3). All experiments were independently repeated at least once with consistent results. ns: not significant, *p* < 0.05 (*), *p* < 0.001 (***) and *p* < 0.0001 (****).

Next, we evaluated the effect of (S)-MG132 on AHR across liver cell models (HepG2, Hep3B, and Hepa1c1c7) at both the mRNA and protein levels. Although (S)-MG132 rescued AHR from degradation in all three cell lines, (S)-MG132 differentially affected the *AHR* mRNA expression (Figure 2A). In HepG2 cells, treatment with 10 µM (S)-MG132 for 6 h significantly increased *AHR* mRNA levels by 2.6-fold, whereas only a modest increase (1.2-fold) was observed in Hep3B cells. In contrast, *AHR* mRNA levels were markedly reduced to 0.4-fold in Hepa1c1c7 cells following 10 µM (S)-MG132 treatment for 6 h. These findings indicated cell line-dependent transcriptional responses to proteasome inhibition. Despite these transcriptional changes, AHR protein abundance did not exhibit a corresponding pattern in all cell lines (Figure 2B). (S)-MG132 (5–20 µM) had a statistically insignificant effect on AHR protein levels in HepG2 cells but significantly reduced AHR protein levels in both Hep3B (0.7-fold) and Hepa1c1c7 (0.5-fold) cells. The increased amount of *AHR* mRNA in HepG2 and Hep3B cells by (S)-MG132 did not translate into more AHR protein abundance, suggesting that additional mechanism(s) could be controlling the final AHR protein levels. However, reduction of the *AHR* mRNA in Hepa1c1c7 cells by (S)-MG132 resulted in a reduction of the AHR protein levels, even though (S)-MG132 rescued AHR from protein degradation. This reduction in Hepa1c1c7 cells was mediated through inhibition of proteasomal degradation since another proteasome inhibitor, lactacystin (10–30 µM) showed a similar pattern of suppression of *AHR* mRNA and protein levels in Hepa1c1c7 cells (Figure 2C). To determine whether (S)-MG132 affected *AHR* mRNA stability in Hepa1c1c7 cells, we performed Act-D experiment to inhibit gene transcription. The *AHR* mRNA decay was monitored over time (0–6 h). Results showed that *AHR* mRNA levels declined similarly in the presence or absence of (S)-MG132 (Figure 2D), suggesting that (S)-MG132 does not alter its mRNA stability but likely suppresses the *AHR* gene transcription. Collectively, (S)-MG132 has opposing effects on the *AHR* mRNA in human and mouse hepatoma cells, and its effect on the AHR protein levels is contrary to its effect on the mRNA level in human hepatoma cells. Although AHR undergoes proteasomal degradation in mouse and human hepatoma cells, inhibition of proteasomal degradation does not increase the AHR protein levels in HepG2, Hep3B, and Hepa1c1c7 cells.

### 2.3. Effect of (S)-MG132 on Global Protein Synthesis in Mouse and Human Hepatoma Cells

**Figure 3:**
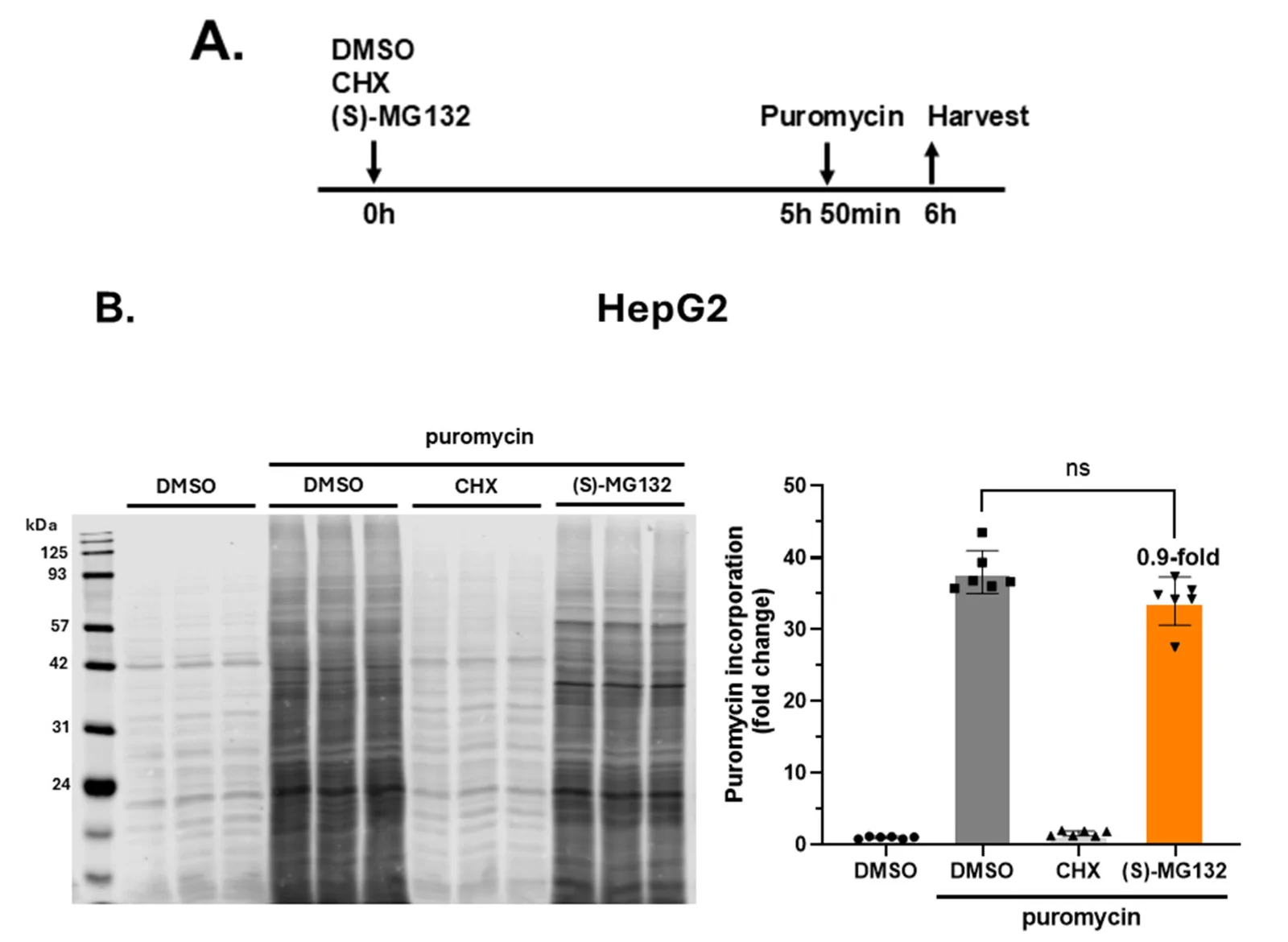

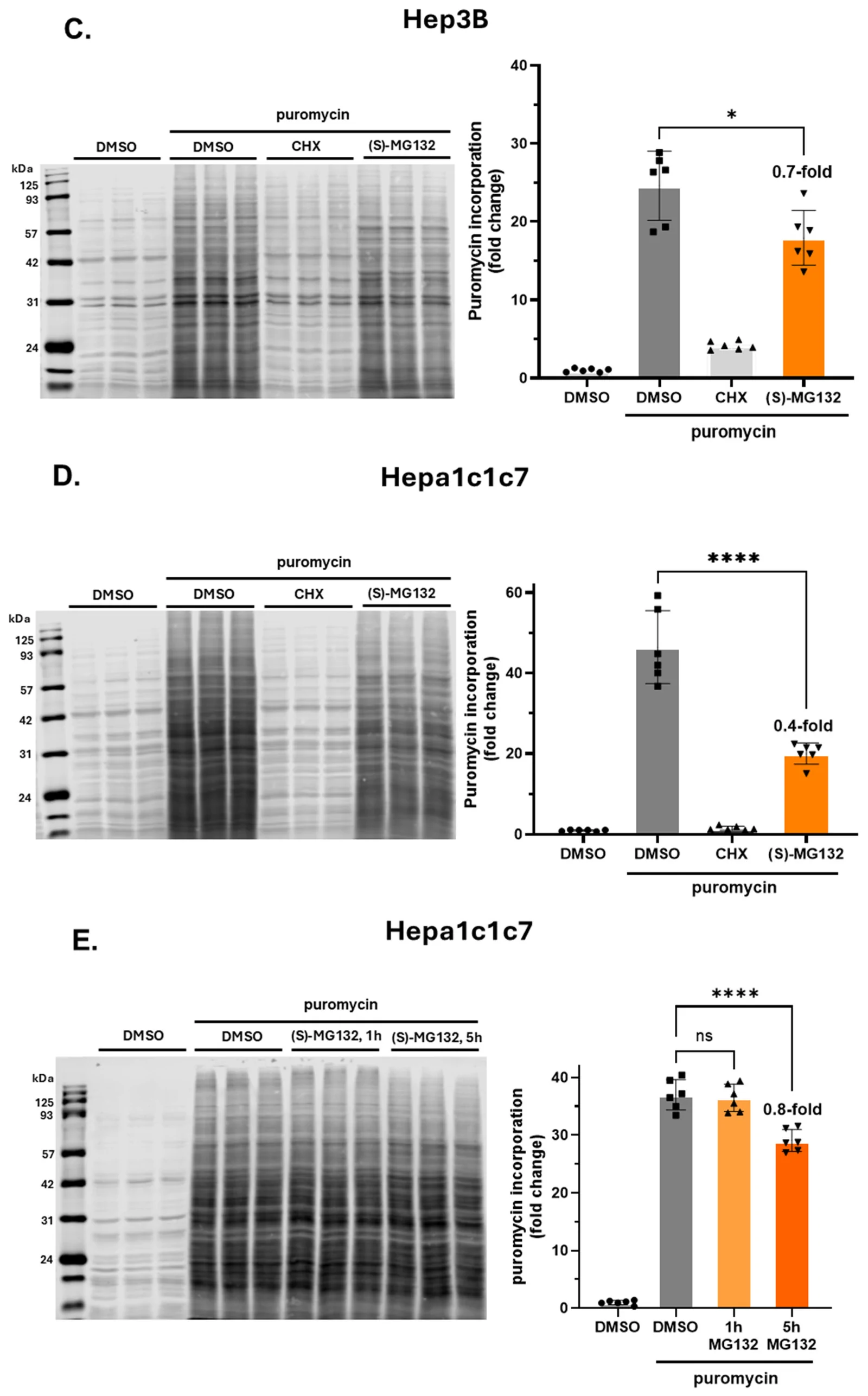
Effects of CHX and MG132 on global protein synthesis in human and mouse hepatoma cells. (**A**) Timeline of the puromycin incorporation (SUnSET) assay. Cells were treated with DMSO, 50–100 µg/mL of CHX, or 10 μM (S)-MG132 for 6 h at 37 °C. Puromycin (10 μg/mL) was added during the final 10 min prior to harvest. Global protein synthesis was assessed in HepG2 (**B**), Hep3B (**C**), and Hepa1c1c7 (**D**) cells using the SUnSET assay. Representative anti-puromycin Western blots and corresponding quantification are shown. Puromycin incorporation was quantified and expressed as fold change relative to the DMSO control with no puromycin. CHX served as a positive control for translational inhibition. HepG2 cells were treated with 100 μg/mL CHX, whereas Hep3B and Hepa1c1c7 cells were treated with 50 μg/mL CHX. (**E**) Time-dependent effects of (S)-MG132 on global protein synthesis in Hepa1c1c7 cells. Cells were treated with 10 μM (S)-MG132 for either 1 h or 5 h at 37 °C, and puromycin was added during the final 10 min before harvest. Representative anti-puromycin Western blots and corresponding quantification are shown. Data are presented as means ± SD. Statistical significance was determined using unpaired two-tailed Student’s *t*-test (**B**–**D**) and one-way ANOVA followed by a multiple-comparisons test (**E**). Each data point represents an independent biological replicate. All experiments were independently repeated at least once with consistent results. ns: not significant, *p* < 0.05 (*) and *p* < 0.0001 (****).

To address whether the suppressive effect of (S)-MG132 on the AHR protein levels is related to protein synthesis, global protein synthesis was assessed using the puromycin incorporation assay, which is also known as the SUnSET assay [35] (Figure 3A). This method measures nascent protein synthesis through the incorporation of puromycin into newly synthesized polypeptides, which are detected using an anti-puromycin antibody. Treatment with CHX (50–100 µg/mL) markedly reduced puromycin incorporation in all three cell lines, confirming that effective inhibition of global protein synthesis was achieved (Figure 3B–D). In HepG2 cells, treatment with 10 µM (S)-MG132 for 6 h reduced global protein synthesis to 0.9-fold, but this reduction was not statistically significant (Figure 3B). In contrast, 10 µM (S)-MG132 significantly reduced puromycin incorporation in Hep3B and Hepa1c1c7 cells to 0.7-fold and 0.4-fold, respectively, after 6 h (Figure 3C,D), indicating that (S)-MG132 suppresses global protein synthesis. Notably, the effect of (S)-MG132 on puromycin incorporation differed between mouse and human hepatoma cells, with a significantly greater reduction observed in mouse than in human hepatoma cells, indicating that a cell line-dependent regulation of global protein synthesis coupled to proteasome inhibition. To further evaluate the timing of this effect in Hepa1c1c7 cells, early treatment (1–5 h) with 10 µM (S)-MG132 showed that (S)-MG132 did not reduce puromycin incorporation after 1 h, but significantly reduced puromycin incorporation modestly after 5 h (to 0.8-fold) (Figure 3E), suggesting that there is a delayed response of the (S)-MG132-mediated translational inhibition which develops many hours after treatment in mouse Hepa1c1c7 cells. In summary, our data supported that (S)-MG132 inhibits global protein synthesis in mouse (Hepa1c1c7) but to a lesser extent in human (HepG2 and Hep3B) hepatoma cells, which translates into lowering of the AHR protein levels.

### 2.4. Effect of (S)-MG132 on the Mouse AHR Promoter

**Figure 4:**
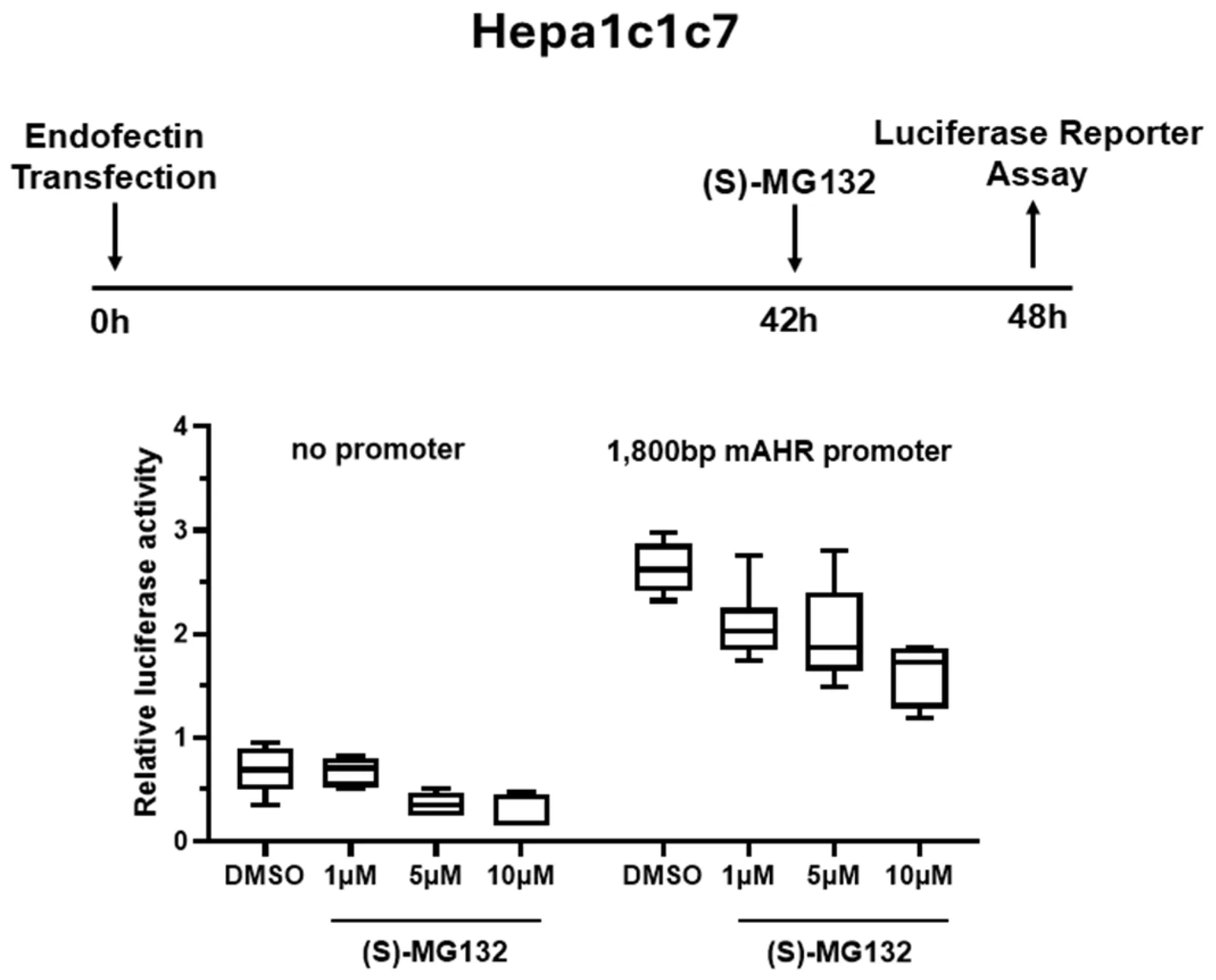
Effect of (S)-MG132 on mouse AHR promoter. Hepa1c1c7 cells were transfected with either a promoterless pGL3-basic luciferase control reporter plasmid or a reporter plasmid containing an 1800-bp fragment upstream of the mouse *AHR* transcription start site. At 42 h after transfection, cells were treated with vehicle (DMSO) or (S)-MG132 (1, 5, or 10 μM) for 6 h at 37 °C. Luciferase activity was measured at 48 h after transfection and expressed as relative luciferase activity to DMSO control. Data represent at least six biological replicates (*n* ≥ 6) in each condition obtained from two independent experiments.

To address how (S)-MG132 reduced the *AHR* mRNA levels in Hepa1c1c7 cells, we performed the mouse AHR promoter-driven luciferase experiments in the presence or absence of (S)-MG132. We cloned 1800 bp upstream of the *AHR* transcription start site into a promoterless luciferase plasmid (pGL3-basic) to investigate the underlying mechanism of this (S)-MG132-dependent suppression of gene transcription. Unfortunately, the cloned reporter plasmid was very weak, with only <4-fold increase of luciferase activity when compared to the control promoterless reporter plasmid (Figure 4). (S)-MG132 (10 µM) only suppressed 40% of the luciferase activity whereas 60% suppression of luciferase activity was observed when the promoterless plasmid was used. We conclude that this 1800 bp fragment does not contain the regulatory elements responsible for the (S)-MG132 suppression of the *AHR* gene transcription.

### 2.5. Overexpression of the HaloTag Fusion of Mouse AHR to Investigate the (S)-MG132 Effect on AHR Protein Degradation

**Figure 5:**
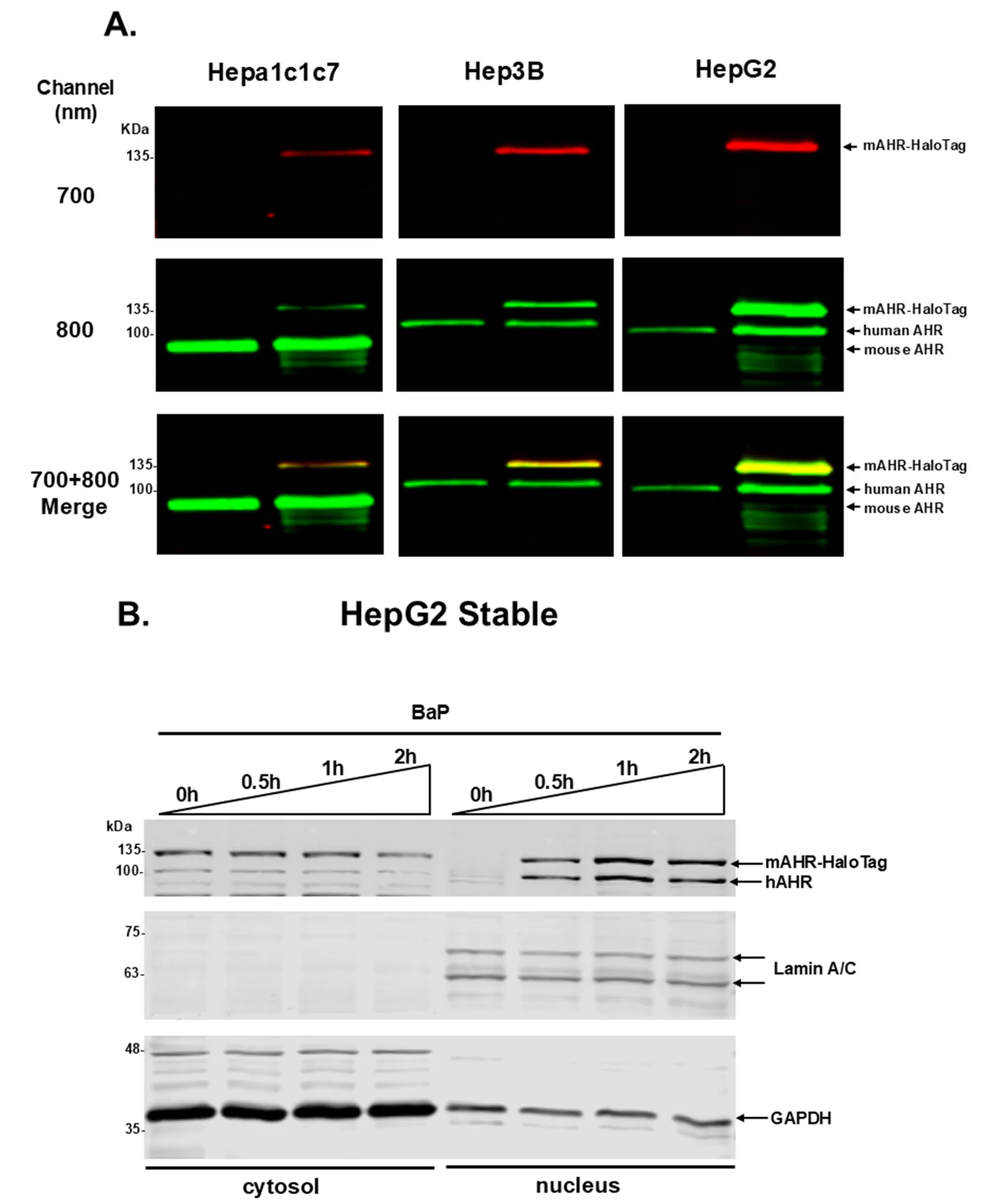

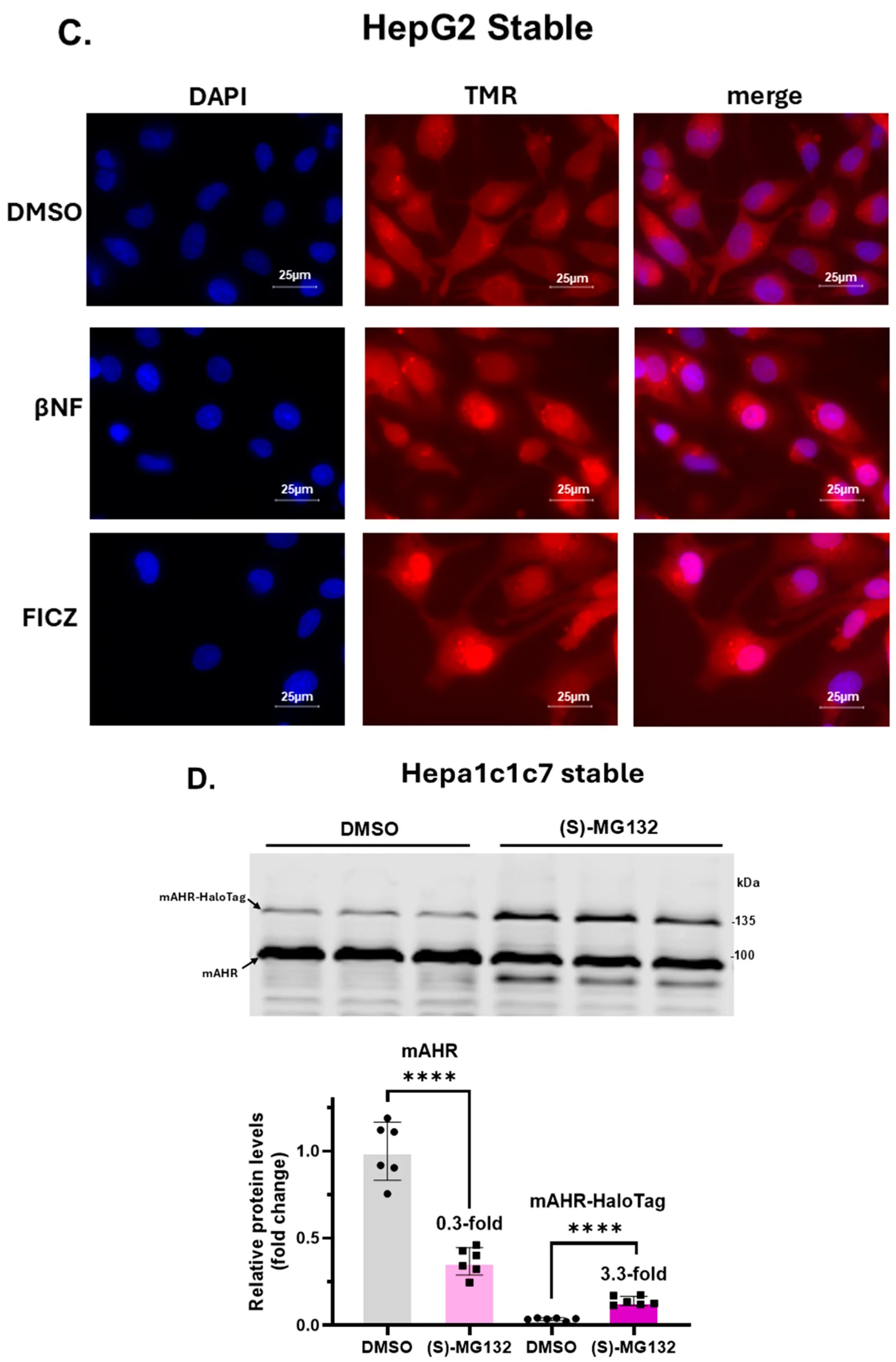

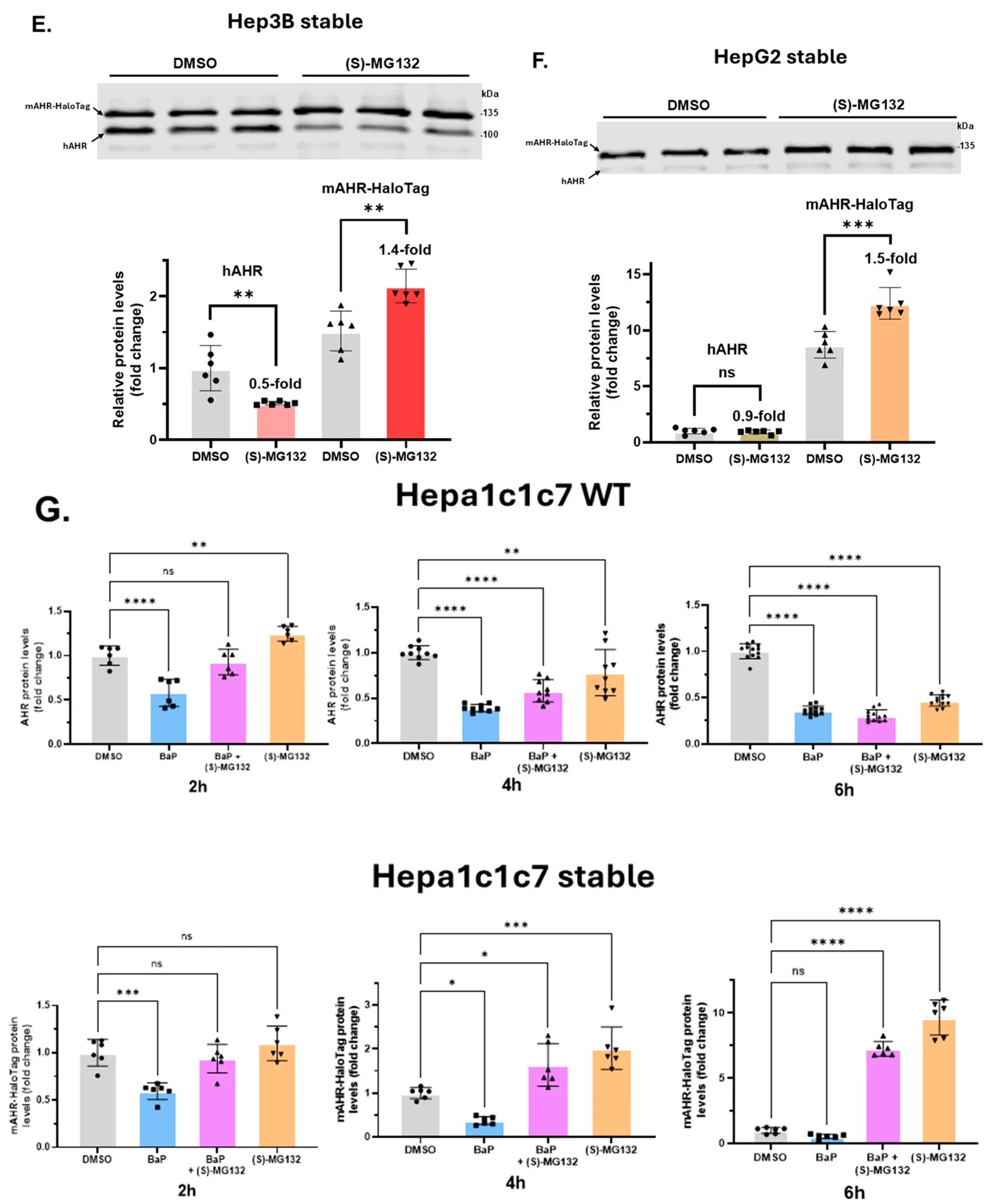
Characterization and regulation of mouse AHR-HaloTag expression in hepatoma cells. (**A**) Validation of exogenous mouse AHR-HaloTag (mAHR-HaloTag) expression in Hepa1c1c7, Hep3B, and HepG2 cells. Cell lysates were analyzed using the HaloTag ligand ViaTag and an anti-AHR antibody (SA210). The 700 nm channel detected ViaTag-labeled mAHR-HaloTag (red, upper panel), whereas the 800 nm channel detected AHR by SA210 (green, middle panel). Merged images are shown to confirm the identity of the exogenous mouse AHR-HaloTag band (bottom panel). mAHR-HaloTag was detected at approximately 135 kDa whereas cellular mouse or human AHR was detected at 96 and 100 kDa, respectively. (**B**) HepG2 cells stably expressing mAHR-HaloTag were treated with BaP (5 μM) for 0, 0.5, 1, or 2 h at 37 °C, followed by subcellular fractionation. mAHR-HaloTag and cellular AHR (hAHR) contents in the cytosolic and nuclear fractions were assessed by Western blot analysis. Lamin A/C and GAPDH were used as nuclear and cytosolic markers, respectively. (**C**) βNF and FICZ induce nuclear translocation of mAHR-HaloTag in HepG2 stable cells. Cells were labeled with 5 μM TMR HaloTag ligand, followed by treatment with DMSO, 5 μM βNF, or 100 nM FICZ for 30 min at 37 °C. Representative fluorescence images show DAPI-stained nuclei (blue, left panels), TMR-labeled mAHR-HaloTag (red, middle panels), and merged images (right panels). Both βNF and FICZ treatment increased the nuclear localization of mAHR-HaloTag compared with the DMSO control. Images were acquired using a Keyence BZ-X700 fluorescence microscope with a 100× oil-immersion objective. Scale bars, 10 μm. Hepa1c1c7 (**D**), Hep3B (**E**), and HepG2 (**F**) cells stably expressing mAHR-HaloTag were treated with DMSO or (S)-MG132 (10 μM) for 6 h at 37 °C. Cellular AHR (mAHR and hAHR) and mAHR-HaloTag protein levels were normalized to the corresponding DMSO-treated control which was arbitrarily set to 1. (**G**) Hepa1c1c7 wild-type (WT) and mAHR-HaloTag stable cells were treated with DMSO, BaP (5 μM), BaP (5 μM) in combination with (S)-MG132 (10 μM), or (S)-MG132 (10 μM) alone for 2, 4, and 6 h. Cellular mouse AHR and mAHR-HaloTag protein levels normalized to DMSO control which was arbitrarily set to 1. Data are presented as means ± SD. Statistical significance was determined using an unpaired two-tailed Student’s *t*-test (**D**–**F**), and one-way ANOVA followed by a multiple-comparisons test (**G**). Each data point represents an independent biological replicate (*n* ≥ 3). Experiments were independently repeated at least once with consistent results. ns: not significant, *p* < 0.05 (*), *p* < 0.01 (**), *p* < 0.001 (***) and *p* < 0.0001 (****).

Next, we performed additional experiments to confirm that (S)-MG132 suppressed AHR protein degradation. We examined AHR protein degradation without the influence of the AHR promoter and the 5′- and 3′-UTR sequences of the *AHR* mRNA. To do that, a plasmid carrying the fusion of HaloTag and the full-length mouse AHR cDNA downstream of the CMV promoter was stably integrated into the genome of hepatoma cells via lentiviral infection. Transcription of this mouse AHR-HaloTag fusion gene, which expresses HaloTag at the C-terminus (mAHR-HaloTag), is driven by the CMV promoter but not the endogenous mouse AHR promoter. In addition, this *mAHR-HaloTag* mRNA lacks the 5′- and 3′-UTR sequences of the endogenous mouse *AHR* mRNA which may affect the rate of protein synthesis. Thus, the cellular level of this AHR fusion protein should be governed solely by protein degradation. Following antibiotic selection, stable cell lines expressing the mAHR-HaloTag protein were generated. Expression of this HaloTag fusion protein was verified using the HaloTag ligand ViaTag together with the anti-AHR antibody SA210 (Figure 5A). The ViaTag signal specifically identified the mAHR-HaloTag, whereas SA210 detected both cellular AHR and mAHR-HaloTag. The amount of mAHR-HaloTag expression when compared to the cellular AHR were 3%, 150%, and 870%, respectively, in Hepa1c1c7, Hep3B, and HepG2 stable cells. To address the functionality of mAHR-HaloTag, we performed subcellular fractionation experiment and fluorescence microscopy using HepG2 stable cells. Among the three stable cell lines, HepG2 stable cells exhibited the highest mAHR-HaloTag protein expression and were therefore selected for subsequent subcellular fractionation and nuclear localization studies. Both the cellular AHR and mAHR-HaloTag translocated into the nucleus 30 min after exposure to an AHR ligand BaP (5 µM) (Figure 5B). Two other typical AHR ligands, namely βNF (5 µM) and FICZ (100 nM), triggered nuclear localization of mAHR-HaloTag after 30 min exposure (Figure 5C). BaP was not used for fluorescence imaging because it interfered with the DAPI staining of the nucleus under our experimental conditions. Collectively, this mouse AHR, in the form of a HaloTag fusion protein, behaves like the human AHR in HepG2 cells.

In contrast to the cell type-dependent effects of AHR following (S)-MG132 treatment in Figure 1, we observed that mAHR-HaloTag significantly accumulated in all three stable cell lines upon (S)-MG132 treatment (Figure 5D–F), confirming that AHR undergoes proteasomal degradation. The cellular AHR in all three stable cell lines behaved like the wild-type cells with content suppression by (S)-MG132, suggesting that the process of stable cell generation did not alter the regulation of cellular AHR expression. However, upon (S)-MG132 treatment, mAHR-HaloTag accumulated in all three stable cell lines (1.4- to 3.3-fold), validating that AHR undergoes proteasomal degradation in all three hepatoma cell lines, particularly in Hepa1c1c7 cells. These findings confirm that reduction of the AHR protein levels in Hepa1c1c7 cells by (S)-MG132 in Figure 2B is primarily due to the suppression of AHR protein synthesis.

Next, we used Hepa1c1c7 wild type and stable cells to address the ligand-dependent proteasomal degradation of cellular AHR and mAHR-HaloTag (Figure 5G). Proteasomal degradation of AHR was blocked by (S)-MG132 after 2 h of BaP exposure, as expected, but not after 6 h. This observation could be explained by our previous results showing that (S)-MG132 suppressed global protein synthesis after 5 h so that the net effect on AHR protein content was suppression instead of accumulation. Similarly, mAHR-HaloTag undergoes proteasomal degradation upon BaP treatment (2–6 h) in Hepa1c1c7 stable cells. However, (S)-MG132 reversed the BaP-dependent proteasomal degradation of mAHR-HaloTag at 2, 4, and 6 h, and (S)-MG132 alone increased the mAHR-HaloTag protein levels in a time-dependent manner, strongly supporting the mAHR-HaloTag protein undergoes proteasomal degradation in the presence or absence of an exogenous AHR ligand.

### 2.6. Effect of (S)-MG132 on HepG2 and Hep3B Spheroids

**Figure 6:**
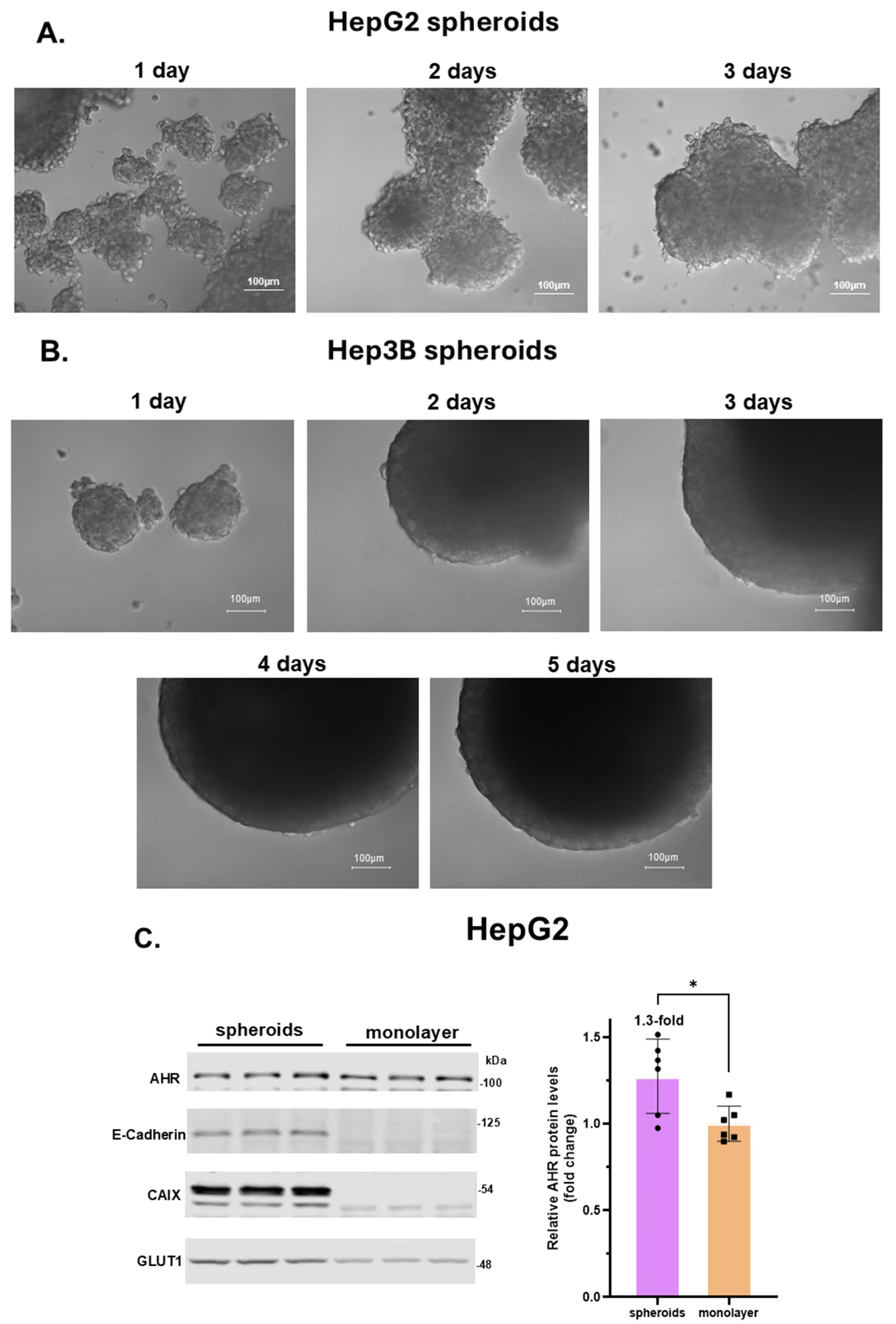

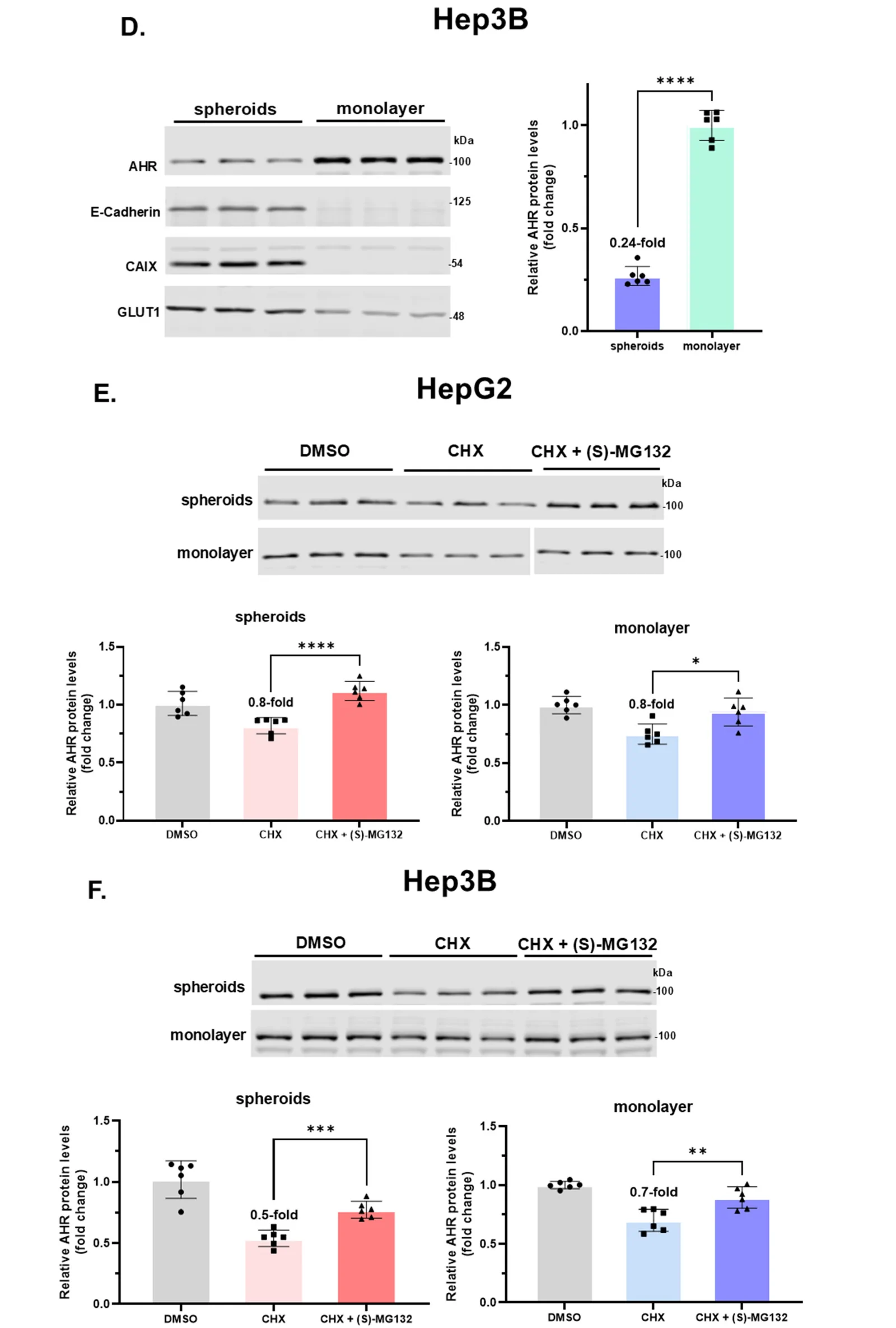
Formation of HepG2 and Hep3B spheroids and regulation of AHR protein expression by CHX and (S)-MG132. (**A**,**B**) Representative bright-field images of HepG2 (**A**) and Hep3B (**B**) cells cultured in ultra-low attachment (ULA) plates. HepG2 spheroids were cultured for 1–3 days, whereas Hep3B spheroids were cultured for 1–5 days. Cells progressively aggregated and compacted over time, forming larger spheroid-like structures. All images were acquired using the same objective under identical imaging conditions (20× objective). Scale bar = 100 μm. AHR protein expression in HepG2 (**C**) and Hep3B (**D**) spheroids was compared with monolayer counterparts by Western blot analysis. E-cadherin, CAIX, and GLUT1 were assessed as markers associated with spheroid formation and the three-dimensional tumor-like phenotype. AHR protein levels were normalized to the corresponding monolayer cultures. HepG2 (**E**) and Hep3B (**F**) spheroids and corresponding monolayer cultures were treated with DMSO, CHX (100 μg/mL for HepG2 and 50 μg/mL for Hep3B), or a combination of CHX and 10 μM (S)-MG132 for 6 h at 37 °C. AHR protein levels were assessed by Western blot and normalized to the DMSO control which was arbitrarily set to 1. HepG2 monolayer images in (**E**) were from the same immunoblot with few irrelevant lanes being cut off between CHX and CHX + (S)-MG132. Data are presented as means ± SD. Statistical significance was determined using an unpaired two-tailed Student’s *t*-test. Each data point represents an independent biological replicate (*n* = 6). All experiments were independently repeated at least once with consistent results. *p* < 0.05 (*), *p* < 0.01 (**), *p* < 0.001 (***) and *p* < 0.0001 (****).

To determine whether the regulation of AHR expression observed in monolayer cultures could be preserved in a three-dimensional model, HepG2 and Hep3B spheroids were generated using ultra-low attachment (ULA) plates. Cells progressively formed compact spheroid-like aggregates over 3–5 days (Figure 6A,B). Both HepG2 and Hep3B spheroids showed upregulation of proteins (E-cadherin, CAIX/CA9, and GLUT1) that were indicative of changing morphology to become more like a tumor which exhibits restriction of oxygen and nutrient availability (Figure 6C,D). When compared to the wild type counterparts, the AHR protein contents were higher in HepG2 spheroids (1.3-fold) but much lower in Hep3B spheroids (0.24-fold), suggesting that there might be some cell line-specific mechanisms that affect the AHR content differently. Similar to the HepG2 monolayer culture, CHX treatment for 6 h significantly reduced AHR protein levels in HepG2 spheroids to 0.8-fold of the DMSO control, whereas co-treatment with (S)-MG132 attenuated the CHX-induced reduction of the AHR protein levels (Figure 6E). These findings demonstrate that proteasome-mediated AHR degradation is preserved in HepG2 spheroids, which closely resembles the conventional 2D culture. However, there was more degradation of the AHR protein in Hep3B spheroids than in monolayer culture with 50% and 70%, respectively, remaining 6 h after CHX treatment (Figure 6F). MG132 was able to rescue the reduction of the AHR protein levels in both cases, suggesting that AHR undergoes proteasomal degradation in Hep3B spheroids similar to the 2D culture. Collectively, proteasomal degradation of AHR without exogenous ligand treatment is observed in human hepatoma spheroids with in vivo relevance.

## 3. Discussion

AHR cellular contents are governed by its synthesis and degradation. Without exogenous ligand, AHR undergoes lysosomal degradation in several human cell lines, namely HeLa, MDA-MB-468, and A549 [33,34,36]. Surprisingly, AHR is continuously undergoing degradation, not primarily by lysosomal degradation, but via the 26S proteasome in hepatoma cell lines. Although the AHR protein half-life has been reported to have a wide range of 7 [37] to 28 h [38] in Hepa1c1c7 cells, we could not observe reproducible AHR protein reduction after 6 h in our CHX experimehnts, which is most likely due to experimental variations which make discernment of small changes occurring in 6 h difficult. We eventually captured the AHR protein degradation when we extended the treatment time to beyond 10 h with an exchange of media with treated reagents every 4 h, ensuring that reagents were active during the treatment period. In our hands, the turnover rate of AHR is relatively long in hepatoma cells, with a half-life much longer than 12 h, when compared to, for example, MDA-MB-468 cells, of which AHR has a half-life of 6–8 h [36]. Interestingly, AHR is not degraded linearly over time in mouse Hepa1c1c7 and human HepG2 hepatoma cells but reaches a plateau above 50% of its content, suggesting that 50% of AHR cellular contents may be necessary to sustain cell growth. However, this plateau phenomenon is not present in human hepatoma Hep3B cells. Rather, the AHR protein levels were consistently reduced only after 6 hours of CHX treatment. This phenomenon is intriguing and we speculate that possibly a proteasome-sensitive AHR degrader is at play gradually after CHX treatment in Hep3B cells.

AHR typically translocates into the nucleus within 30–60 min after ligand binding, followed by proteasomal degradation of AHR which occurs shortly after that. This is supported by our subcellular fractionation and fluorescence staining results showing that AHR gets into the nucleus of HepG2 cells upon 30 min of ligand exposure. Inhibition of proteasomal degradation by MG132 rescues AHR from degradation, which increases the AHR protein levels. For all our experiments, we specified the use of (S)-MG132 because we did not observe any rescue of AHR from degradation when (R)-MG132 was used. It has been reported that this rescue occurred after 4–6 h of TCDD treatment in Hepa1c1c7 cells [32]. However, we could not replicate the rescue in 4–6 h; instead, we were able to rescue AHR from proteasomal degradation in 2 h instead. The 2 h window is consistent with the ligand-triggered proteasomal degradation, which should occur shortly after translocation of AHR into the nucleus. Since we used BaP as the ligand, we cannot rule out the possibility that the timing difference is due to the differences of AHR response to TCDD versus BaP. Six hours after ligand addition, co-treatment with (S)-MG132 shows an even lesser amount of AHR protein, which is due to the delayed inhibition of AHR protein synthesis by (S)-MG132. These (S)-MG132 effects are clear in mouse Hepa1c1c7 cells, but not in human HepG2 and Hep3B cells, since reduction of the *AHR* mRNA levels and pronounced inhibition of global protein synthesis by (S)-MG132 only occur in mouse but not in human hepatoma cells. Nonetheless, (S)-MG132 causes measurable inhibition of general protein synthesis in Hep3B and HepG2 cells, and the extent of inhibition mirrors the reduction of AHR protein levels in these cells. When both synthesis and degradation of AHR are inhibited in human (HepG2 and Hep3B) hepatoma cells for 6 h by (S)-MG132, the net effect is reduction of AHR protein levels, implying that human AHR protein levels are more sensitive to the changes in its synthesis than degradation within that period. In addition to reduction of general protein synthesis by (S)-MG132 in Hepa1c1c7 cells, the *AHR* mRNA levels are clearly reduced (by half) upon (S)-MG132 treatment. This reduction of the *AHR* mRNA is likely mediated through suppression of the *AHR* gene transcription but not affecting the *AHR* mRNA stability. We postulate that a factor that is sensitive to proteasomal degradation suppresses the *AHR* gene transcription by binding to a regulatory element of the AHR promoter. This putative regulatory element does not reside in 1800 bp upstream of the transcription start site since we did not observe any MG132-dependent suppression of luciferase expression when this 1800 bp fragment was cloned upstream to the luciferase gene.

We generated the HaloTag fusion of mouse AHR (mAHR-HaloTag) driven by the CMV promoter to uncouple the (S)-MG132 effect on the mouse AHR promoter and the regulatory elements on the 5′- and 3′-UTRs. Expression of mAHR-HaloTag in hepatoma cells via lentiviral infection allows us to confirm that AHR undergoes proteasomal degradation without exogenous ligand treatment. We characterized this mAHR-HaloTag fusion protein and showed that the AHR moiety is functional with regards to ligand-activated nuclear localization. We observed that mAHR-HaloTag clearly accumulates upon (S)-MG132 treatment, validating that AHR undergoes proteasomal degradation, and the net reduction of AHR protein levels by (S)-MG132 is mediated through actions on the AHR promoter in Hepa1c1c7 cells, not just via inhibition of general protein synthesis. The precise mechanism of how (S)-MG132 inhibits AHR protein synthesis remains unclear.

There are some limitations of this study. Since we used immortalized cancer cell lines, the differences we observed among human and mouse hepatoma cells might not be reflecting AHR in mouse and human livers. Although we successfully generate spheroids that resemble the tumor microenvironment better than the 2D culture, interpretations of our results must be cautioned since our spheroids do not contain any normal hepatocytes, stromal, and immune cells as expected in the liver tissue.

We cultured HepG2 and Hep3B spheroids in an effort to address the in vivo relevance of ligand-independent AHR protein degradation. HepG2 and Hep3B spheroids exhibit similar effects to our 2D cell culture in terms of the rescue of AHR protein degradation by MG132, further validating that AHR undergoes proteasomal degradation in human hepatoma cells. It is conceivable that the AHR abundance might be altered when cells form spheroids since it mimics human tissues. Hepatoma spheroids are known to change protein expression patterns in response to oxygen and nutrient deprivation to their hypoxic core [39]. The AHR signaling, such as the AHR-dependent CYP1A1 induction [40], can be different when compared between HepG2 monolayer and spheroids. Our spheroids exhibit an epithelial phenotype with increased expression of E-cadherin, and oxygen and nutrient deprivation with increased expression of CAIX and GLUT1. It is unclear why AHR contents are slightly increased in HepG2 spheroids but markedly decreased in Hep3B spheroids.

## 4. Materials and Methods

### 4.1. Reagents and Antibodies

BaP, puromycin, PMSF, leupeptin, polybrene, puromycin dihydrochloride, βNF, and actinomycin D were purchased from Sigma-Aldrich (St. Louis, MO, USA). CQ, (S)-MG132 and CHX were purchased from Cayman Chemical (Ann Arbor, MI, USA). Dimethyl sulfoxide (DMSO) was purchased from Invitrogen (Carlsbad, CA, USA). pCMV-dR8.2 dvpr was a gift from Bob Weinberg (Addgene plasmid #8455; RRID: Addgene_8455). Purified plasmid was generated using the Zymopure miniprep or maxiprep kit (Zymo Research, Irvine, CA, USA). The mouse AHR-HaloTag (mAHR-HaloTag) expression construct (pReceiver-lv129) and endofectin transfection reagent were purchased from GeneCopoeia (Rockville, MD, USA). TRI Reagent and a Direct-zol RNA miniprep kit were purchased from Zymo Research (Irvine, CA, USA). MMLV high-performance reverse transcriptase was purchased from Epicentre (Madison, WI, USA). iTaq SYBR green supermix was purchased from Bio-Rad (Hercules, CA, USA). PCR master mix was purchased from Promega (Madison, WI, USA). FBS (HyClone), DMEM (HyClone), Opti-MEM, and Pierce BCA assay agents were purchased from Thermo Fisher Scientific (Waltham, MA, USA). GlutaMAX-I and penicillin–streptomycin were purchased from Invitrogen (Carlsbad, CA, USA). Rabbit anti-AHR polyclonal antibody (SA210) was purchased from Enzo Life Sciences (Farmingdale, NY, USA). Mouse anti-AHR monoclonal antibody (A-2) and mouse anti-E-cadherin monoclonal antibody (G-10; sc-8426) were purchased from Santa Cruz Biotechnology (Dallas, TX, USA). Mouse anti-puromycin monoclonal antibody (MABE343) was purchased from Merck Millipore (Burlington, MA, USA). Rabbit anti-GLUT1 polyclonal antibody (NB110-39113) and rabbit anti-CA9 polyclonal antibody (NB100-417) were purchased from Novus Biologicals (Centennial, CO, USA). Donkey anti-rabbit and donkey anti-mouse secondary antibody conjugated with IRDye 700CW or IRDye 800CW, Revert 700 Total Protein Stain, and nitrocellulose membrane were purchased from LI-COR Bioscience (Lincoln, NE, USA). The HaloTag^®^ TMR Ligand (G825A), Wizard^®^ SV Gel and PCR Clean-Up System kit, and Dual-Glo^®^ Luciferase Assay System kit were purchased from Promega (Madison, WI, USA). The synthetic mouse AHR promoter fragments (Fragment 1A: 1–1250 bp and Fragment 2: 1234–1800 bp) were purchased from Twist Bioscience (South San Francisco, CA, USA). NEBuilder^®^ HiFi DNA Assembly Master Mix (E2621S) was purchased from New England Biolabs (Ipswich, MA, USA).

### 4.2. Cell Culture

Hepa1c1c7, Hep3B and HepG2 cells were purchased from ATCC, and were authenticated by ATCC. AD293 cells were purchased from Agilent Technologies (Santa Clara, CA, USA). All the cell lines (including all stable cell lines) were cultured in DMEM supplemented with 10% FBS, 1% penicillin–streptomycin, and 1% GlutaMAX-I at 37 °C, 5% CO_2_.

### 4.3. Preparation of Whole Cell Extract and Western Blot Analysis

Cells were trypsinized, collected by centrifugation at 400× *g* for 5 min, washed once with cold PBS, and centrifuged again at 400× *g* for 5 min to obtain cell pellets. Cell pellets were lysed in lysis buffer (25 mM HEPES pH7.4, 0.4 M KCl, 1 mM EDTA, 1 mM DTT, 10% glycerol, 1% NP40, 1 mM PMSF, and 2 µg/mL leupeptin) by 3 cycles of freeze/thaw. Lysed cells were kept on ice for 30 min and then centrifuged at 16,000× *g* for 15 min at 4 °C. The supernatants were used as whole-cell extracts. Total protein concentrations were measured by BCA assay. Whole cell extracts (20 µg) were separated by 12% SDS-PAGE and then transferred to a nitrocellulose membrane via the wet transfer method (constant amp, 300 mA, 120 min). LI-COR Revert 700 Total Protein Stain was used to stain all proteins on the membrane after transfer. The intensity of all proteins in a sample was determined by selecting the whole lane at 700 nm channel using the LICOR Image Studio software (version 5.2.5). Non-specific binding was blocked in a blocking buffer (PBS, 0.1% Tween-20, and 5% BSA) for 1 h. The primary antibodies and their dilution are as follows: 1:2000 for anti-AHR (SA210); 1:500 for mouse anti-AHR(A-2), anti-GLUT1, anti-CAIX and anti-E-Cadherin. After washing with PBST (PBS and 0.1% Tween20) 5 times, the nitrocellulose membrane was incubated in 1:10,000 dilution of donkey secondary antibody conjugated with IRDye 800 CW. Results were detected using a LI-COR Odyssey CLx imaging system (Lincoln, NE, USA). The Western signals of all samples at 800 nm channel were normalized by the corresponding total protein stain intensities using the LICOR Image Studio software.

### 4.4. RNA Extraction and RT-qPCR

Total RNA was extracted from cultured cells using TRI Reagent (Zymo Research) and an RNA miniprep kit (Direct-zol) according to the manufacturer’s recommendations. cDNA was reverse transcribed from 1 µg of RNA using MMLV high-performance reverse transcriptase (Epicentre) into a final volume of 20 µL of cDNA solution, of which 1 µL was used as the qPCR template. qPCR was performed with iTaq SYBR green supermix (Bio-Rad) on a Bio-Rad CFX Connect real-time PCR operating system according to the following protocol: 95 °C for 2 min, 40 cycles of 95 °C for 15 s, and 60 °C for 1 min. Relative gene expression was analyzed by the 2^−∆∆Cq^ method, and *18S* rRNA was used as an internal control for normalization. The primer sequences were as follows: *18S* forward: 5′-CGCCCCCTCGATGCTCTTAG-3′ and reverse: 5′-CGGCGGGTCATGGGAATAAC-3′; *Rps18* forward: 5′-AACACCGAAAAATCGAGGAGTG-3′ and reverse: 5′-GAGCTTGGGTTTATCCTTTGGT-3′; human *AHR*: forward: 5′-ACATCACCTACGCCAGTCGC-3′ and reverse: 5′-TCTATGCCGCTTGGAAGGAT-3′; mouse *AHR*: forward: 5′-GCCCTTCCCGCAAGATGTTAT-3′ and reverse: 5′-CAGGGGTGGACTTTAATGCAA-3′.

### 4.5. Puromycin Incorporation (SUnSET) Assay

Global protein synthesis was assessed using the SUnSET assay. At the last 10 min of cell treatment, puromycin was added to the culture medium at a final concentration of 10 μg/mL. Cells were then collected and lysed for Western blot analysis. Puromycin-labeled nascent polypeptides were detected using an anti-puromycin antibody (MABE343). Puromycin signal intensity was quantified using LICOR Image Studio software and then normalized by the total protein stain.

### 4.6. Generation of Mouse AHR-HaloTag Overexpression Stable Cell Lines Using Lentivirus

Lentiviral particles were generated in AD293 cells. Briefly, AD293 cells (7 × 10^5^) were seeded in a 25 cm^2^ flask containing 5 mL of DMEM supplemented with 10% FBS and 2 mM GlutaMAX-I but lacking penicillin and streptomycin. After incubation at 37 °C and 5% CO_2_ overnight, cells reached 50–80% confluence. The medium was replaced with fresh antibiotic-free medium. AD293 cells were then transfected using 10 µL of endofectin transfection reagent with a total of 5 µg plasmids which included 2.5 µg of cloned plasmid containing the mouse AHR-HaloTag fusion cDNA (which is pReceiver-Lv129-mouse AHR full-length plasmid from GeneCopoeia, a lentiviral vector carrying the full length mouse AHR cDNA with the HaloTag cDNA at the 3′ end), 1.875 µg of pCMV-dR8.2 dvpr packaging plasmid, and 0.625 µg of pCMV-VSV-G envelope plasmid. Fresh complete medium was added 15 h after transfection. After 24 h, the medium containing lentiviral particles was collected and stored at 4 °C. Another 5 mL of fresh complete medium was added to the cells, and the medium containing lentiviral particles was harvested again after an additional 24 h. The combined medium was centrifuged at 400× *g* for 5 min to remove AD293 cells, and the supernatant was used for infection. For stable cell generation, Hepa1c1c7, HepG2, and Hep3B cells were seeded in 25 cm^2^ flasks to reach 50–70% confluence the next day. The medium was replaced with fresh complete medium containing 8 µg/mL of polybrene, and 3 mL of lentivirus-containing medium carrying the mouse AHR-HaloTag expression construct was added to each flask. After 24 h, the medium was replaced with fresh complete medium containing 2 µg/mL of puromycin for stable cell selection. AHR-HaloTag expression was confirmed by Western blot analysis and HaloTag ligand ViaTag labeling.

### 4.7. Construction of the Mouse AHR Promoter Luciferase Reporter Plasmid

Two mouse AHR promoter overlapping fragments upstream of the transcription start site were synthesized commercially (Fragment 1A: 1–1250 bp and Fragment 2: 1234–1800 bp). The fragments were amplified by PCR, separated by agarose gel electrophoresis, and purified using standard gel extraction procedures. PCR amplification was performed using Q5 high-GC enhancer and Q5 DNA polymerase (New England Biolabs) in a 25 μL reaction containing 5 μL of 5× Q5 reaction buffer, 0.5 μL of dNTP mix (10 mM each), 1.25 μL of each forward and reverse primer (10 μM), 1 ng of template DNA, 5 μL of Q5 high-GC enhancer, 0.25 μL of Q5 DNA Polymerase, and nuclease-free water to a final volume of 25 μL. The primer sequences were as follows: Fragment 1A: forward: 5′-TACGCGTGCTAGCCCGAA-3′ and reverse: 5′-GGAGTGCTGAATTTCTGTTA-3′; Fragment 2: forward: 5′-CAGAAACTCAGCACTCCTG-3′ and reverse: 5′-GCAGATCTCGAGCCCCAC-3′. PCR cycling conditions consisted of an initial denaturation at 98 °C for 30 s, followed by 35 cycles of 98 °C for 20 s, 55 °C for 15 s, and 72 °C for 2 min 40 s, with a final extension at 72 °C for 2 min. PCR products were separated by agarose gel electrophoresis, excised, and purified using the Wizard^®^ SV Gel and PCR Clean-Up System (Promega) according to the manufacturer’s instructions. The purified promoter fragments were assembled into the pGL3-Basic luciferase reporter vector using NEBuilder^®^ HiFi DNA Assembly master mix (E2621S, New England Biolabs) according to the manufacturer’s instructions. The assembled plasmids were transformed into NEB 5 alpha chemically competent cells (C2987). Positive colonies were expanded, and plasmid DNA was purified for luciferase reporter assay. The cloned reporter plasmid was designated as mAHR promoter-pGL3.

### 4.8. Luciferase Reporter Assay

Hepa1c1c7 cells were seeded in 96-well plates at a density of 5 × 10^4^ cells per well and allowed to attach overnight. Cells were co-transfected with 200 ng of either mAHR promoter-pGL3 or empty pGL3-basic plasmid together with 50 ng of Renilla-CMV plasmid using 0.6 µL of endofectin transfection reagent (GeneCopoeia) per well. Six hours after transfection, complete growth medium was added to the wells. The following day, the medium was replaced with fresh complete medium. Forty-two hours after transfection, cells were treated with (S)-MG132 at final concentrations of 1, 5, or 10 µM for 6 h at 37 °C. Luciferase activity was measured 48 h after transfection using the Dual-Glo^®^ Luciferase assay system (E2920, Promega). Firefly luciferase activity was normalized to Renilla luciferase activity to determine relative promoter activity.

### 4.9. Spheroid Formation and Culture

HepG2 and Hep3B cells were seeded at 2 × 10^4^ cells per well in a Nunclon Sphera ultra-low attachment (ULA) 12-well plate containing complete culture medium and maintained under standard culture conditions (37 °C, 5% CO_2_). Spheroid formation was monitored over time. HepG2 spheroids were cultured for up to 3 days, whereas Hep3B spheroids were cultured for 5 days to obtain sufficient amount of cells for analysis. At the indicated time points, spheroids were collected directly from the ULA plate into centrifuge tubes without enzymatic dissociation. The collected spheroids were pelleted by centrifugation and proceeded for protein extraction and Western blot analysis.

### 4.10. Subcellular Fractionation

HepG2 cells stably expressing mAHR-HaloTag were cultured in 75 cm^2^ flasks until approximately 90% confluent and treated with BaP (5 μM) for 0, 0.5, 1, or 2 h. Following treatment, cells were detached using TrypLE Express (Thermo Fisher Scientific, Grand Island, NY, USA), washed with ice-cold PBS, and collected by centrifugation at 400× *g* for 5 min at 4 °C. The cell pellets were resuspended in 250 μL of ice-cold hypotonic lysis buffer containing 25 mM HEPES, pH 7.4, 5 mM KCl, 0.5 mM MgCl_2_, 1 mM DTT, 0.5% NP-40, 1 mM PMSF, and 2 μg/mL of leupeptin, followed by tumbling rotation at 60 rpm for 15 min at 4 °C. Samples were then centrifuged at 3000× *g* for 2 min at 4 °C, and the supernatants were collected as the cytosolic fractions. The remaining pellets were washed once with ice-cold hypotonic lysis buffer and twice with ice-cold PBS, followed by resuspension in 100 μL of ice-cold nuclear extraction buffer containing 25 mM HEPES, pH 7.4, 350 mM NaCl, 10% sucrose, 0.05% NP-40, 1 mM DTT, 1 mM PMSF, and 2 μg/mL of leupeptin. Nuclear proteins were extracted by tumbling rotation at 60 rpm for 1 h at 4 °C. Samples were subsequently centrifuged at 16,000× *g* for 15 min at 4 °C, and the supernatants were collected as the nuclear fractions. Protein concentrations of the cytosolic and nuclear fractions were determined using a BCA protein assay.

### 4.11. HaloTag TMR Labeling and Fluorescence Imaging

HepG2 cells stably expressing mAHR-HaloTag were seeded onto poly-L-lysine-coated glass coverslips in 12-well plates at a density of 7 × 10^4^ cells per well and cultured overnight at 37 °C, 5% CO_2_. Cells reached approximately 40–60% confluence at the time of treatment. The following day, cells were incubated with 5 μM TMR HaloTag ligand for 30 min at 37 °C in the dark. Following labeling, cells were gently washed twice with pre-warmed culture medium and incubated in fresh medium for an additional 30 min at 37 °C. Cells were then treated with DMSO, 10 μM βNF, or 100 nM FICZ for 30 min at 37 °C. Following treatment, cells were washed once with PBS and then fixed with 4% paraformaldehyde for 20 min at room temperature in the dark. Cells were subsequently washed three times with PBS. Coverslips were mounted onto glass slides using SlowFade Diamond Antifade Mountant (Thermo Fisher Scientific, Waltham, MA, USA) with DAPI and protected from light. Fluorescence images were acquired using a Keyence BZ-X700 fluorescence microscope (KEYENCE Corporation, Osaka, Japan) with a 100× oil-immersion objective to assess the subcellular localization and ligand-induced nuclear translocation of mAHR-HaloTag.

### 4.12. Statistical Analysis

GraphPad Prism 11 software (La Jolla, CA, USA) was utilized for statistical analysis. Two-tailed unpaired *t*-test, multiple *t*-tests corrected with the Holm–Sidak method for multiple comparisons, and one-way and two-way (or mixed-model) ANOVA with Sidak, Tukey or Dunnett tests for multiple comparisons were used to determine statistical significance with * *p* < 0.05, ** *p* < 0.01, *** *p* < 0.001, and **** *p* < 0.0001; ns, not significant.

## 5. Conclusions

AHR of hepatoma cells undergoes proteasomal degradation without exogenous ligand treatment in 2D culture and spheroids. Inhibition of proteasomal degradation suppresses AHR protein synthesis in mouse and human hepatoma cells.

## Data Availability

The original contributions presented in this study are included in the article. Further inquiries can be directed to the corresponding author.

## Abbreviations

The following abbreviations are used in this manuscript:

AHR	aryl hydrocarbon receptor
AHRR	aryl hydrocarbon receptor repressor
ARNT	aryl hydrocarbon receptor nuclear translocator
Act-D	actinomycin-D
BaP	benzo[a]pyrene
CHX	cycloheximide
βNF	β-naphthoflavone
CMV	cytomegalovirus
CQ	chloroquine
DAPI	4′,6-diamidino-2-phenylindole
DMEM	Dulbecco’s modified Eagle medium
DMSO	dimethyl sulfoxide
FBS	fetal bovine serum
FICZ	6-formylindolo[3,2-b]carbazole
HSP90	heat shock protein 90
PBS	phosphate-buffered saline
PPARγ	peroxisome proliferator-activated receptor gamma
RT-qPCR	reverse transcription-quantitative polymerase chain reaction
SDS-PAGE	sodium dodecyl sulfate-polyacrylamide gel electrophoresis
SUnSET	surface sensing of translation
TiPARP	TCDD-inducible poly(ADP-ribose) polymerase
TMR	tetramethylrhodamine
UTR	untranslated region
WT	wild type
XAP2	hepatitis B virus-associated protein 2
